# Macrophage activation and polarization in post-infarction cardiac remodeling

**DOI:** 10.1186/s12929-017-0322-3

**Published:** 2017-02-07

**Authors:** Aleksandra Gombozhapova, Yuliya Rogovskaya, Vladimir Shurupov, Mariya Rebenkova, Julia Kzhyshkowska, Sergey V. Popov, Rostislav S. Karpov, Vyacheslav Ryabov

**Affiliations:** 1 Cardiology Research Institute, Tomsk National Research Medical Center, Russian Academy of Sciences, Tomsk 111a Kievskaya Street, 634012 Tomsk, Russian Federation; 2 National Research Tomsk State University, 36 Lenin Avenue, 634050 Tomsk, Russian Federation; 3 Siberian State Medical University, 2 Moscovsky trakt, 634055 Tomsk, Russian Federation; 4 University of Heidelberg, 1-3 Theodor-Kutzer Ufer, 68167 Mannheim, Germany

**Keywords:** Myocardial infarction, Inflammation, Macrophages, Monocytes, Remodeling, Heart failure

## Abstract

Adverse cardiac remodeling leads to impaired ventricular function and heart failure, remaining a major cause of mortality and morbidity in patients with acute myocardial infarction. It have been shown that, even if all the recommended therapies for ST-segment elevation myocardial infarction are performed, one third of patients undergoes progressive cardiac remodeling that represents morphological basis for following heart failure. The need to extend our knowledge about factors leading to different clinical scenarios of myocardial infarction and following complications has resulted in a research of immuno-inflammatory pathways and molecular activities as the basis for post-infarction remodeling. Recently, macrophages (cells of the innate immune system) have become a subject of scientific interest under both normal and pathological conditions. Macrophages, besides their role in host protection and tissue homeostasis, play an important role in pathophysiological processes induced by myocardial infarction. In this article we summarize data about the function of monocytes and macrophages plasticity in myocardial infarction and outline potential role of these cells as effective targets to control processes of inflammation, cardiac remodeling and healing following acute coronary event.

## Background

Recent data suggest that modern methods of interventional and pharmacological therapies have already implemented their potential to limit infarct size, reduce mortality and improve contractile function in patients during and after acute myocardial infarction [1, 2]. Cardiac remodeling following myocardial infarction is a process of alterations in cardiac geometry, function and structure, which is considered to be a universal response to an increased wall stress or loss of the viable myocardium [3, 4]. It leads to impaired ventricular function and heart failure, remaining a major cause of mortality and morbidity [5–8].

During the last decade both experimental and clinical studies have been identifying several modified and unmodified predictors of adverse cardiac remodeling [9–12]. Obviously, not all experimental data can be extrapolated to the clinical data. It is critical to underscore that reperfusion time is a cornerstone factor determining post-infarction cardiac remodeling [1, 4, 13]. The response to ischemic injury in infarct area and in the remote viable myocardium has a definite time sequence. However, different severity grades of cardiac remodeling develop. But at the same time, the processes of cardiac healing and remodeling, even in similar clinical scenarios, under equal conditions such as infarct size, location, clinic period prior to treatment, therapy strategy, age - occur in different ways [4, 14]. Bolognese [13] et al. and others [4] have shown that, even if all the recommended therapies for ST-segment elevation myocardial infarction are performed, one third of patients undergo progressive cardiac remodeling that represents morphological basis for following heart failure.

**Fig. 1:**
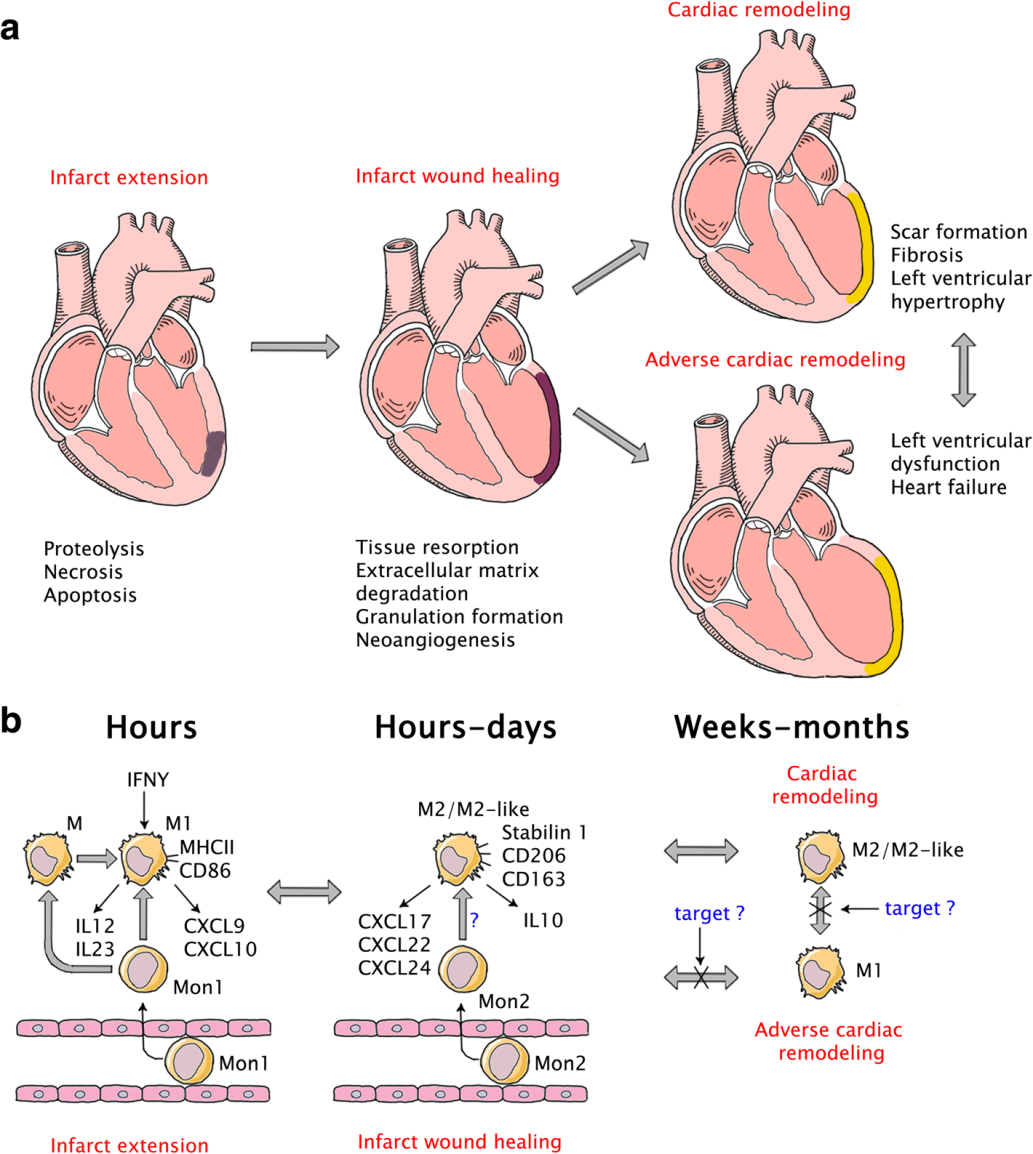
**a** Paradigms dealing with mechanisms of cardiac remodeling after myocardial infarction. In the early phase of injury, ventricular remodeling is an effect of infarct expansion; in late phase it involves reactive myocyte hypertrophy, interstitial fibrosis and left ventricular dilatation. Changes of the extracellular collagen matrix in infarct heart play an important role in cardiac remodeling A huge number of endogenous factors affect the extracellular collagen matrix in different ways, causing degradation or synthesis of its components. **b** Cardiomyocyte necrosis triggers an activation of innate immune system and a cascade of inflammatory pathways. In response to ischemic injury monocytes are recruited from the bone marrow and spleen to the heart and become monocyte-derived macrophages. Macrophages produce pro- and anti-inflammatory factors, promote resorption of cellular debris, regulation of granulation tissue formation and neoangiogenesis. The search of therapeutic target, which is able to prevent, limit or reverse adverse cardiac remodelingg is one of the most important and complicated tasks of modern cardiology

In the era of reperfusion treatment two paradigms dealing with mechanisms of cardiac remodeling after myocardial infarction have been formed [15] (Fig. 1a). According to the first paradigm, in the early phase of injury, ventricular remodeling is an effect of infarct expansion (process of myocardial wall thinning and dilatation); and in the later phase, it is secondary in regard to surviving myocardium reconstruction involving reactive myocyte hypertrophy, interstitial fibrosis and left ventricular dilatation [16, 17]. The second paradigm is based on the idea, that changes of the extracellular collagen matrix in both infarct and non-infarct zones of myocardium play a major role in cardiac remodeling [18, 19].

Over the last decade the improvement and development of medical technology have led to rise of attention, in particular, to the second paradigm. It has become clear that a huge number of endogenous factors affect the extracellular collagen matrix in different ways, causing degradation or synthesis of its components. There are number of hormones, renin-angiotensin-aldosterone system, different cytokines, matrix metalloproteinases and their tissue inhibitors [20, 21]. Interactions and regulation of these molecules take part in left ventricular remodeling process and conform to development of cardiac healing, which is, also, the complex process of well-defined and time-dependent continuous and overlapping events. Despite of the fact that over the last 30 years, achievements in pharmacological and interventional treatment have reduced mortality in patients with acute myocardial infarction, there is still no effective method influencing process of myocardial healing [22, 23]. Nowadays, the search of therapeutic target, which is able to prevent, limit or reverse adverse cardiac remodeling and interrupt the development of left ventricular dilatation, is still one of the most important and complicated tasks of modern cardiology.

Suggested paradigms of cardiac remodeling became the reason of wide use of anti-fibrotic strategies. ACE inhibition [24], angiotensin receptor antagonism [25], mineralocorticoid blockade [26] and HMG-Coa-reductase inhibition [27] reduce the development of progressive interstitial and perivascular fibrosis, and contribute to beneficial cardiac remodeling. However, some patients undergo progressive heart failure despite of the anti-fibrotic treatment administration. Obviously, more effective prevention of progressive remodeling is compulsory [28].

The need to extend our knowledge about factors leading to different clinical scenarios of myocardial infarction and following complications has resulted in a research of immuno-inflammatory pathways and molecular activities as the basis for post-infarction remodeling.

Cardiomyocyte necrosis triggers an activation of innate immune system and a cascade of inflammatory pathways. Besides being some kind of «warning system», innate immunity is a complex molecular network which is sensitive to different danger signals defined during cell necrosis and degradation of extracellular matrix components. Recently, macrophages (cells of the innate immune system) have become a subject of scientific interest under both normal and pathological conditions. Macrophages are an integral part of innate immune response. They are equipped with a set of pathogen recognition receptors, which can activate phagocytosis of pathogens and the secretion of cytokines and chemokines. They present antigens on their cell surface by major histocompatibility complex II (MHC II) and interact closely with the adaptive immune system. Monocytes/macrophages and resident macrophages are key participants of inflammatory response, they produce pro- and anti-inflammatory factors, promote resorption of cellular debris, regulation of granulation tissue formation and neoangiogenesis. In many ways, they determine cardiac remodeling and healing after myocardial infarction through secretion of proteases, growth factors, influence cardiomyocyte apoptosis and proliferation.

Thus, we summarize data about the function of monocytes and macrophages plasticity in myocardial infarction and outline potential role of these cells as effective targets to control processes of inflammation, cardiac remodeling and healing following acute coronary event.

## Monocytes and macrophages in health and disease

Monocytes are white blood cells developed in bone marrow from progenitor cells. From the bone marrow monocytes go into blood. Under homeostatic conditions they circulate in blood for 1–3 days [29]. Then monocytes migrate into different organs, where they become tissue macrophages and, also, give rise to dendritic cells. Macrophages are one of major effectors of homeostasis in many organs, including brain, liver, adipose tissue, lymphatic system, gastro-intestinal tract [30]. They realize functions such as phagocytosis, cytokine production, antigen presentation.

Besides, monocytes can be attracted to the tissues by infectious and inflammation conditions, when they begin to play a key role in innate immune defense and are involved in tissue remodeling and repair. Nowadays functions of monocytes/macrophages and their subsets are actively investigated in different clinical settings: cancer, infectious, autoimmune, liver, kidney and cardiovascular diseases.

### Origin of cardiac macrophages

It is known that a heart consists of several cell types, including cardiomyocytes, cardiac fibroblasts, endothelial and smooth muscle cells. In addition to these basic types of cells, macrophages are also found in the mammalian heart.

Previously believed, that blood-derived monocytes are the only source of macrophages in the heart. This idea has been disputed by recent work demonstrating the yolk sac and fetal liver as a common sources of macrophages in adult tissues [31, 32]. According to these findings, cardiac macrophages have their origin, at least to some extent, in the yolk sac (YS). Initially YS-derived macrophages show a common signature, later their distinct phenotype and functions are formed by local environment of the resident tissue [33]. Tissue macrophages perform homeostatic functions and immune control. Removal of damaged cardiomyocytes and pro-inflammatory effects of macrophages are their routine functions and has been shown in a number of articles [30, 34]. Nevertheless, their role in inflammatory processes in the heart needs to be defined.

It remains unknown, for how long YS-derived macrophages reside in adult tissues. In organs, such as the brain and the liver, these cells are not substituted at constant state and can persist independently of hematopoietic stem cells [35]. However, replacement of tissue macrophages by bone marrow-derived monocytes is possible in a specific environment (e.g., intestine) or as a result of bacterial infections [36]. In fact, the number of YS-derived resident macrophages in the mouse heart also seems to be non-permanent and declines with age. As mice grow older cardiac macrophages rate of proliferation decreases and becomes insufficient to preserve the resident macrophage pool [37]. Furthermore, resident macrophages in the heart are lost as a consequence of myocardial infarction. So, they need to be substituted through monocyte recruited from the circulation, or by local proliferation of resident cells. Further experimental evidence will be required to define the quantitative contribution of circulation monocytes to the cardiac macrophage pool during aging and myocardial injury conditions. Finally, it is still unknown to what extent effector functions of monocyte-derived macrophages are distinct from resident macrophages [38]. Undoubtedly, the findings will have implications for our understanding of cardiac homeostasis and disease.

### Activation and plasticity of monocytes/macrophages

One of the key monocyte/macrophages features, influencing their functions, is their phenotypic and functional plasticity [39, 40]. Under physiological and pathological conditions these cells are able to modulate functional and morphological characteristics, activate effector functions. Thus, monocyte/macrophage “activation” process gives rise to cell phenotypes with distinct and determined roles [41].

Currently, repeating the classification of T-helper cells, which separates the cells on Th1 and Th2 types, macrophages are divided into two different subsets [42]: classically activated or interferon-γ (γ-IFN) mediated M1 macrophages exhibiting a strong bactericidal activity and secreting large amounts of pro-inflammatory mediators, and alternatively activated or interleukin-4/interleukin-13(IL-4/IL-13) mediated M2 macrophages [43] showing a high phagocytic activity and expressing IL-10, decoy type II receptors, antagonist of receptor IL-1 (IL-1ra) [44].

### Classically activated macrophages

The classical activation occurs after macrophage contacts with activated T-helper 1 lymphocyte (Th-1). Production of Th-1 cells is stimulated by viruses and some bacterias, primarily intracellular pathogens. The stimulus for their differentiation from immature effector T-lymphocytes (Th-0) is secretion of IL-12 by dendritic cells and IFN-γ by natural killers (NK-cells). After activation Th-cell begins to secrete a certain range of cytokines, including IFN-γ. IFN-γ alone or together with lipopolysaccharide or cytokines such as tumor necrosis factor (TNF) and granulocyte-macrophage colony-stimulating factor (GM-CSF) were first found mediators of macrophage classical activation [45].

Initially, activated macrophage, meeting with the pathogen, phagocytes it, exposes to processing, loads proteins got in result of antigen processing to the molecules of MHC II and presents complex of MHC II and protein to its surface [46]. This process is accompanied by the production of pro-inflammatory cytokines – IL-12, IL-23, IL-27, TNF, chemokines – CXCL9, CXCL10, CXCL11 and also by expressing surface markers - cluster of differentiation (CD) 40, CD80, CD86 [27]. It results in increase of nitric oxide, reactive oxygen species, proteolytic enzymes release, including metalloproteinase (MMP)-1, -2, -7, -9 and -12 which degrade collagen, elastin, fibronectin and other extracellular matrix components [47]. Reactive oxygen species cause cytotoxic effects of activated macrophages [28, 48]. Described mechanisms are aimed to host protection, mediate resistance against intracellular parasites and tumors, but, at the same time, during a long uncontrolled or sudden intensive secretion conditions, they might be the cause of cellular and extracellular components destruction. Thus, classically activated macrophages secrete pro-inflammatory cytokines, promote the development of inflammation, extracellular matrix degradation and apoptosis.

### Alternatively activated macrophages

Mechanism of macrophage alternative activation is following. One of the first found sufficient signals to maintain the macrophage activity were IL-4 and IL-13, secreted by T-helper 2 lymphocytes (Th-2) [49]. It is now known that other mediators can also drive alternative activation. For example, IL-21 [50], IL-33 [51], IL-34 [52].

Alternatively activated macrophages are characterized by expressing high levels of IL-10, IL-1ra, decoy type II receptors [53], mannose, scavenger and galactose-type receptors. These cells secrete the cytokines CCL17, CCL22, CCL24 [54, 55], transforming growth factor-β (TGF-β) [36], surface markers, such as CD163 [27], stabilin-1 [56]. TGF-β, effecting fibroblasts, enhances their ability to produce extracellular matrix components. The cytokines such as platelet derived growth factor, insulin like growth factor and TGF-β increase cell proliferation and stimulate angiogenesis. An important role in angiogenesis plays vascular endothelial growth factor. All these molecules are produced by alternatively activated macrophages [57]. Thus, M2 macrophages demonstrate immune-regulatory and anti-inflammatory properties; contribute to tissue remodeling, angiogenesis and tumor progression [58]. It is necessary to mention, that modern classification system subdivides M2 macrophages into M2a, M2b, M2c cells [59]. Classification the M2 phenotype into subtypes highlights general properties and activation mechanisms of these cells. M2a and M2c macrophages are crucial for promoting the adaptive immune response, whereas suppression and regulation of inflammation and immunity are mostly regulated by M2b cells [60].

In this way, function of alternatively activated macrophages, unlike classically activated cells, is devoted to repair process and resolution of inflammation. Different stimuli like glucocorticoids, immunoglobulin complexes, TGF-β, IL-10 generate formation of M2-like phenotypes that show some but not all the properties of alternatively activated macrophages [25]. So, presence of macrophages with overlapping M1/M2 characteristics suggests about remarkable plasticity of these cells.

## Cardiac macrophages subsets in post-infarction cardiac remodeling

There are three phases of the immune response to myocardial ischemia: very early (hours), an early (hours-days) and a late (weeks-months) phase [61]. Replacement of the necrotic myocardium with granulation tissue takes place during the initial inflammatory phase. The following phases result in fibrosis and scar formation.

### Monocytes/macrophages response to myocardial ischemia

In response to ischemic injury monocytes are recruited from the bone marrow and spleen to the heart and become monocyte-derived macrophages [21] (Fig. 1b). Nowadays functional properties of resident cardiac macrophages are not clarified yet [43]. Mostly, it presents technical difficulties in performance of specific genetic manipulations to characterize functional contribution of resident cells. One of the devised methods of macrophage function assessment is encapsulation of clodronate with liposomes and its delivery into macrophages via phagocytosis, resulting in macrophage depletion [62]. Several studies have shown cardioprotective functions of cardiac macrophages in a mouse myocardial cryoinjury model [63–65]. Application of the clodronate liposome decreased vascular endothelial growth factor (VEGF) [52] and TGF-β [53] expression, causing delayed myocardial debris removal and impairment of neoangiogenesis. Thus, histological and cellular analysis demonstrates that controlled recruitment and coordinated activation of monocytes/macrophages are necessary for optimal infarct healing because of promotion of cellular debris resorption and apoptotic cells, degradation of extracellular matrix components, regulation of granulation tissue formation, and neoangiogenesis (Fig. 1b). In clinic, disbalance of these processes is observed in progressive thinning of infarct area, chamber dilatation and systolic dysfunction, or, on the other hand, in increase myocardial stiffness, impaired relaxation and progressive diastolic dysfunction.

### Monocytes/macrophages subsets

Although monocyte/macrophages phenotype diversity is not fully characterized, there is binary classification of these cells for understanding their functions.

In mice models, blood monocytes were divided into two subsets: Ly6C^hi^ inflammatory monocytes recruited into injured tissues show high levels of expression of the CC chemokine receptor CCR2, whereas expressing low levels of the fractalkine receptor CX3CR1 (Ly-6C^hi^CCR2^hi^CX3CR^low^); and subset of Ly6C^low^CCR2^low/neg^CX3CR1^hi^ monocytes (CD14dimCD16+ in humans) [66].

Initial work suggested that early phase after infarction is dominated by inflammatory Ly6C^hi^ monocytes/macrophages. These cells produce inflammatory cytokines like IL-1β, IL-6 and TNF-α, but, at the same time, realize a cardioprotective function mediating proteolysis and phagocytose cell debris during acute inflammatory phase [21, 67, 68]. In contrast to Ly6C^hi^monocytes, Ly6C^low^monocytes are recruited in the later post-infarct stages and mediate myocardial healing through secretion of anti-inflammatory cytokines and growth factors, such as VEGF and TGF-ß, thus, contribute to myofibroblast activation and neoangiogenesis [28, 55]. In its turn cardiac fibroblasts can recruit monocytes via MCP-1-mediated chemotaxis and adhesion to ICAM-1/VCAM-1, and induce their differentiation to M1 or M2 macrophages [69]. Recently it was shown that M2 polarization in infarcted mouse and human hearts is dependent on macrophage-derived urokinase plasminogen activator [70]. At the same time excess of urokinase plasminogen activator promotes increased fibroblast migration and/or proliferation. Also, hydrogen sulfide (H_2_S), a novel endogenous gasomediator, stimulates M2 macrophage polarization, thus.preventing post-infarction adverse remodeling [71].

It is important to say, that studies devoted to description of mouse cardiac monocyte/macrophages subsets and to refinement of its classification are continuing. One of the recent study have described four subsets of mouse cardiac CD45+CD11b+F4/80+ macrophages [72]. There were used surface markers, including Ly6C, MHCII, CD11c and CCCR. According to the research, Ly6C^hi^ macrophages have been classified as M1 macrophages, and Ly6C^low^ or M2 macrophages were divided into three subsets. It is worth noting that certain of M2 macrophages subsets demonstrated functions that formerly have been considered only as M2 cells activity. For example, one of the M2 macrophage population - MHCII^low^, demonstrated a strong phagocytic activity; another population – MHCII^hi^CD11c^low^CCR2- showed remarkable antigen-presenting function; finally, MHCII^hi^CD11c^hi^CCR2^hi^ macrophages had pro-inflammatory phenotype. In another study Shiraishi et al.[73] identified CD206^+^F4/80^+^CD11b^+^ M2-like macrophages in the murine heart which determined the repair of infarcted heart due their fibroblast activation function. Furthermore, the authors suggested IL-1α and osteopontin as mediators of M2-like macrophage–induced fibroblast activation. The information concerning monocytes/macrophages subsets is summarized in Table 1.

**Table 1:** General characteristics of monocytes/macrophages subsets participating in post-infarction cardiac remodeling

M1 inflammatory monocytes/macrophages (classically activated macrophages)	M2 monocytes/macrophages (alternatively activated macrophages)
Markers	
Ly6C^hi^ CC chemokine receptor CCR2	Ly6C^low^ Fractalkine receptor CX3CR1 MHCII^low^ MHCII^hi^CD11c^low^CCR2- MHCII^hi^CD11c^hi^CCR2^hi^ CD206^+^F4/80^+^CD11b^+^ M2-
Mediators of activation	
Cardiac fibroblasts IFN-γ Granulocyte-macrophage colony-stimulating factor	Cardiac fibroblasts Hydrogen sulfide IL-1α Macrophage-derived urokinase plasminogen activator Osteopontin
Secreted cytokines	
IL-1β IL-6 TNF-α	VEGF TGF-ß
Contributions to cardiac injury and repair	
Mediating of inflammatory phase Proteolysis Phagocytosis of cell debris	Resolution of inflammation Myofibroblast activation Neoangiogenesis

New data once again suggest that there are still a lot of questions about monocytes/macrophages phenotypes and functions under physiological and pathophysiological conditions, including post-infarction cardiac remodeling.

### Monocytes/macrophages plasticity

In that way, the clear categorization of monocyte/macrophages subsets into distinct phases and functions after infarction has been disputing. The developmental relationship between monocyte subsets and macrophage polarization into phenotypically and functionally distinct cells requires further studies. Undoubtedly, monocytes/macrophages have peculiar plasticity that allows them to phenotypically polarize in response to microenvironmental signals according to specific M1 or M2 functional programs. New information, concerning plasticity of these cells and signals that might regulate it, is continuing to accumulate.

It has been reported, that Ly6C^low^ development and survival of monocytes depend on the transcription factor Nr4a1 [74]. Absence of Ly6C^low^ monocytes in Nr4a1-deficient animals does not interrupt the bi-phasic inflammatory response. It might be explained by the concept that Ly6C^low^ cells derive from Ly6C^hi^ monocytes, which demonstrate high plasticity and develop into either pro-inflammatory or anti-inflammatory monocytes [41, 55]. Moreover, there are some findings showing that inflammatory Ly6C^hi^ monocytes are predominantly attracted to the infarct area not only in the first days of infarction but throughout the course of post-infarct remodeling [75]. In another recent study, the authors demonstrated that cardiosphere-derived cells favored heart repair by switching the macrophages from a pro-inflammatory phenotype (M1) into an anti-inflammatory phenotype (M2) [76]. Cardiosphere-derived cells administration decreased M1 macrophages and neutrophils but increased M2 macrophages in the infarcted heart.

Certainly, one of the main signal that can influence the plasticity of monocytes/macrophages is phagocytosis of apoptotic cells after acute myocardial infarction. M1 macrophages are able to change its phenotype into M2 macrophages when recognizing of apoptotic cells occurs [77]. Impaired resolution of inflammation may express in prolonged M1 macrophage activation influencing on myocardial infarction outcomes, including development of adverse cardiac remodeling [55, 78].

Thus, nowadays studies demonstrate cardioprotective role of optimal monocytes/macrophages activation and polarization. That fact allows considering control of recruitment of monocytes/macrophages subpopulations and its plasticity modulation as a new therapeutic approach for the timely influence on post-infarction inflammation and following cardiac remodeling (Fig. 1b).

## New experimental therapeutic approaches for prevention of post-infarction cardiac remodeling

To date modern knowledge about the response of innate immune system and the role of inflammation following myocardial ischemia provides new experimental opportunities in studying and development of new therapeutic targets that could be able to prevent adverse cardiac remodeling and heart failure.

### Macrophage as a therapeutic target for post-infarction myocardial repair

Playing a significant role in the myocardial infarction pathophysiology, monocytes/macrophages are considering as a potential therapeutic target in promoting of myocardial healing. Despite the considerable progress in characterizing factors regulating monocyte/macrophage polarization, nowadays we are at the beginning of understanding the spatiotemporal relationships and functions of the various macrophage subsets in the post-infarction cardiac remodeling [79, 80].

Different stages of monocyte/macrophage vital activity are actively suggested as targets to modulate its activation and polarization. One of the sides is blocking the attraction of inflammatory monocytes. Kempf et al. [81, 82] have shown that in the infarct myocardium stimulation of growth differentiation factor-15 (GDF-15) is occurred, which plays an important role in controlling of inflammatory cell recruitment. GDF-15 is an anti-inflammatory cytokine, which is able to limit the tissue damage by inhibition the attraction of monocytes to the post-infarction inflammation area and reduce episodes of left ventricular rupture. Blocking of the inflammatory monocytes recruitment can be also achieved by exposure to chemokines, which leads to a decrease of circulating inflammatory monocytes (e.g., removal of receptor CCR2), or by inhibiting their implication to ischemic area (e.g., CXC chemokine receptor type 6, CXCR6; macrophage migration inhibitory factor, MMIF). Positive effect on cardiac remodeling can be achieved by reduction of CCR2+ monocytes due to attenuating inflammatory response after myocardial infarction [83, 84]. Moreover, destruction of CXCR6 receptors reduces the amount of CD11b+ cells in the infarcted area and resulting in improved cardiac function and prevention of adverse myocardial remodeling through breaking autophagis response [85]. Inhibition of MMIF affects the apoptosis and other signaling cascades. It is important to note, that plasma MMIF levels are associated with the infarct size and the severity of cardiac remodeling. However we have to remember, that MMIF blockade is not specific, and among other results, it leads to neutrophil migration reduction [86].

Another approach to affect monocyte/macrophage function is the idea of changing their microenvironment. As recently shown, macrophage phenotypes and functions are formed by the microenvironment of the resident organ [87]. In addition, resident macrophages interact with cells of the adaptive immune system. There is a positive effect on wound healing and remodeling due to modulation of monocyte/macrophage differentiation in the setting of myocardial infarction by regulatory T-cells [88]. B-lymphocytes interact with monocytes during cardiac repair process. B-cells contribute to recruitment of Ly6C^hi^ monocytes to the myocardial infarction area by secretion CCL7. Depletion of B-cells leads to decrease of circulating monocytes and inflammatory Ly6C^hi^ monocytes in myocardium and it results in the improvement of contractile function and reduction of infarct area [89]. Though, further experimental and clinical data are required to study exact mechanisms of the cross-reactions between the adaptive immune system and monocytes/macrophages and their therapeutic potential in post-infarction cardiac remodeling.

Finally, resident macrophages can be affected by intracellular signals that, for example, can stimulate the proliferation or apoptosis. Effecting expression of the transcription factor MafB may decelerate macrophage apoptosis during inflammatory conditions [90]. Another example is IL-4, which causes accelerated proliferation of the resident macrophages [91].

### MicroRNAs as targets for prevention of remodeling

Micro ribonucleic acids (miRNAs) are involved in alteration the cellular gene expression response. Significance of miRNAs was shown in multiple processes including tissue repair and injury, immune and inflammatory responses [92, 93]. miRNAs control the development, differentiation, and function of monocytes/macrophages by targeting numerous molecules participating in these processes. Transcription factors playing a crucial role in macrophage polarization are controlled by specific miRNA. The signal transducer and activator of transcription 1 (STAT 1) and interferon-regulatory factor 5 (IRF 5) dictate M1 macrophage polarization [94]. M2 macrophage polarization is directed by STAT 6, peroxisome proliferator-activated receptor-γ (PPARγ), and IRF 4 [94]. For instance, it was demonstrated, that miR-125a-3p and miR-26a-2 are expressed in M1 macrophages, whereas miR-193b, miR-27a, miR-29b-1, miR-132 and miR-222 are expressed in M2 subset [95]. Thus, miRNAs play an important role in regulation of monocytes/macrophages key functions, including their role in post-infarction cardiac remodeling (Table 2) [96].

**Table 2:** miRNAs in regulation of monocytes/macrophages functions during post-infarction cardiac remodeling

miRNAs	Function
miR-146 miR-132 miR-155 miR-9 miR-145 miR-346 miR-21 miR-33	Promotion and resolution of inflammation
miR-210 miR-21 miR-146	Wound healing
miR-21	Phagocytosis
miR-21 miR-126	Apoptosis

miRNAs are important regulators of cellular gene programs in cardiovascular disease, and its targeting may become a useful approach to prevent post-infarction cardiac remodeling [97–99]. Reactivation of the fetal miRNA triggers pathological changes leading to progressive remodeling and heart failure by contribution to alterations of gene expression [100]. Analysis of microRNAs in the heart has to focus not only on cardiomyocytes, but also on fibroblasts, inflammatory cells, endothelial cells, and smooth muscle cells [101]. It has been detected that miRNAs involved both early and late in cardiac healing and remodeling processes during and after myocardial ischemia.

Early after myocardial ischemia reperfusion, miR-21 was localized in the fibroblast-enriched infarct region and may regulate the expression of matrix metalloprotease-2 in the infarct area via its target PTEN (phosphatase and tensin homologue) [102]. Possibly, in that way, up-regulation of miR-21 plays a protective role in the early phase of myocardial infarction [103].

Targeting other miRNAs deregulated after cardiac ischemia was associated with therapeutic benefit: miR-29 appeared to be involved in fibrosis development, whereas miR-92a is an endothelial miR regulating angiogenesis [104, 105] and miR-24 induced endothelial cell apoptosis and inhibited angiogenesis [106]. These effects were mediated through targeting of the endothelium-enriched transcription factor GATA2 and the p21-activated kinase-4. Well-timed treatment after myocardial infarction with an antagomir (synthetic molecule that is complementary to a specific miRNA) against miR-24 reduced left ventricular remodeling in mice and improved cardiac function. In that way, new knowledge concerning mRNAs will be decisive in developing miRNA-based therapies. Considering monocyte/macrophage polarization as an important factor of wound healing after myocardial ischemia, miRNA regulation of myeloid cells may be significantly involved in healing and remodeling after myocardial infarction; however, these investigations are in their beginning stage and have an experimental character [107].

## Conclusion

Despite the progress of experimental studies devoted to the response of innate immune system and the role of inflammation following myocardial infarction, there is no significant advancement in clinical studies. In addition not all experimental data can be extended to the clinical. That is why it is important to verify experimental data concerning monocyte/macrophage activation and polarization in post-infarction cardiac remodeling in clinical material. Identifying effective markers of different monocyte/macrophage subsets in patients with myocardial infarction might be the first step in our knowledge enrichment. The following question includes study of molecules and mechanisms that are able to regulate the balance between M1 and M2 macrophages and become effective targets in prevention of adverse post-infarction cardiac remodeling.

## Abbreviations

ACE: Angiotensin-converting-enzyme
CD: Cluster of differentiation
GDF: Growth differentiation factor
GM-CSF: Granulocyte-macrophage colony-stimulating factor
HMG-Coa-reductase: 3-hydroxy-3-methylglutaryl coenzyme A reductase
ICAM-1: Intercellular adhesion molecule-1
IL: Interleukin
IL-1ra: Antagonist of receptor IL-1
IRF: Interferon-regulatory factor
MCP-1: Monocyte chemoattractant protein-1
MHC II: Major histocompatibility complex II
miRNA: miR - micro ribonucleic acid
MMIF: Macrophage migration inhibitory factor
MMP: Metalloproteinase
NK: Natural killers
PPA-Rγ: Peroxisome proliferator-activated receptor-γ
PTEN: Phosphatase and tensin homologue
STAT: Signal transducer and activator of transcription
TGF-β: Transforming growth factor-β
TNF: Tumor necrosis factor
VCAM - 1: Vascular cell adhesion molecule-1
VEGF: Vascular endothelial growth factor
YS: Yolk sac
γ-IFN: Interferon-γ

## References

[1] Montecucco F, Carbone F, Schindler TH (2016). Pathophysiology of ST-segment elevation myocardial infarction: novel mechanisms and treatment. Eur Heart J.

[2] Ishii H, Amano T, Matsubara T, Murohara T (2008). Pharmacological intervention for prevention of left ventricular remodeling and improving prognosis in myocardial infarction. Circulation.

[3] Opie LH, Commerford PJ, Gersh BJ, Pfeffer MA (2006). Controversies in ventricular remodeling. Lancet.

[4] Ryabov VV, Ryabova TR, Markov VA (2014). Post-infarction heart remodeling in STEMI patients depending on pharmakoinvasive or primary PCI strategy. Eur Heart J: ACC.

[5] Lewis EF, Moye LA, Rouleau JL, Sacks FM, Arnold JM, Warnica JW (2003). Predictors of late development of heart failure in stable survivors of myocardial infarction: the CARE study. J Am Coll Cardiol.

[6] Markov VA, Ryabova TR, Sokolov AA, Dudko VA, Ryabov VV (2002). Dynamics of structural and functional left ventricular parameters with acute myocardial infarction. Kardiologiia.

[7] Mega JL, Braunwald E, Wiviott SD, Bassand JP, Bhatt DL, Bode C (2012). Rivaroxaban in patients with a recent acute coronary syndrome. N Engl J Med.

[8] Roe MT, Ohman EM (2012). A new era in secondary prevention after acute coronary syndrome. N Engl J Med.

[9] Ndrepepa G, Mehilli J, Martinoff S, Schwaiger M, Schömig A, Kastrati A (2007). Evolution of left ventricular injection fraction and its relationship to infarct size after acute myocardial infarction. J Am Coll Cardiol.

[10] Bujak M, Kweon HJ, Chatila K, Li N, Taffet G, Frangogiannis NG (2008). Aging-related defects are associated with adverse cardiac remodeling in a mouse model of reperfused myocardial infarction. J Am Coll Cardiol.

[11] Lok DJ, Klip IT, Lok SI, Bruggink-André de la Porte PW, Badings E, van Wijngaarden J (2013). Incremental prognostic power of novel biomarkers (growthdifferentiation factor-15, high-sensitivity C-reactive protein, galectin-3, and high-sensitivity troponin-T) in patients with advanced chronic heart failure. Am J Cardiol.

[12] Woo JS, Kim WS, Yu TK (2011). Prognostic value of serial global longitudinal strain measured by two-dimensional speckle tracking echocardiography in patients with ST-segment elevation myocardial infarction. Am J Cardiol.

[13] Bolognese L, Neskovic AN, Parodi G, Ha SJ, Kim SY, Bae JH (2002). Left ventricular remodeling after primary coronary angioplasty: patterns of left ventricular dilatation and long-term prognostic implications. Circulation.

[14] Eapen ZJ, Tang WHW, Felker GM, Hernandez AF, Mahaffey KW, Lincoff AM (2012). Defining heart failure end points in ST-segment elevation myocardial infarction trials: integrating past experiences to chart a path forward. Circ Cardiovasc Qual Outcomes.

[15] Jugdutt BI (2003). Ventricular remodeling after infarction and the extracellular collagen matrix: when is enough enough?. Circulation.

[16] Franz S, Bauersachs J, Ertl G (2009). Post-infarct remodeling: contribution of wound healing and inflammation. Cardiovasc Res.

[17] Frangogiannis NG, Smith CW, Entman ML (2002). The inflammatory response in myocardial infarction. Cardiovasc Res.

[18] Lindsey ML, Iyer RP, Jung M, DeLeon-Pennell KY, Ma Y (2016). Matrix metalloproteinases as input and output signals for post-myocardial infarction remodeling. J Mol Cell Cardiol.

[19] Clarce SA, Richardson WJ, Holmes JW (2016). Modifying the mechanics of healing infarcts: is better the enemy of good?. J Mol Cell Cardiol.

[20] Eisen A, Benderly M, Behar S, Goldbourt U, Haim M (2014). Inflammation and future risk of symptomatic heart failure in patients with stable coronary artery disease. Am Heart J.

[21] Nian M, Lee P, Khaper N, Liu P (2004). Inflammatory cytokines and post-myocardial infarction remodeling. Circ Res.

[22] Christia P, Frangogiannis NG (2013). Targeting inflammatory pathways in myocardial infarction. Eur Clin Invest.

[23] Kramer DG, Trikalinos TA, Kent DM, Antonopoulos GV, Konstam MA, Udelson JE (2010). Quantitative evaluation of drug or device effects on ventricular remodeling as predictors of therapeutic effects on mortality in patients with heart failure and reduced ejection fraction: a meta-analytic approach. J Am Coll Cardiol.

[24] The SOLVD Investigators (1991). Effect of enalapril on survival in patients with reduced left ventricular ejection fractions and congestive heart failure. N Engl J Med.

[25] The HEAAL Investigators (2009). Effects of high-dose versus low-dose losartan on clinical outcomes in patients with heart failure (study): a randomized, double-blind trial. Lancet.

[26] The RALES Investigators (1996). Effectiveness of spironolactone added to an angiotensin-converting enzyme inhibitor and a loop diuretic for severe chronic congestive heart failure. Am J Cardiol.

[27] Bauersachs J, Galuppo P, Fraccarollo D, Christ M, Ertl G (2001). Improvement of left ventricular remodeling and function by hydroxymethylglutaryl coenzyme a reductase inhibition with cerivaststin in rats with heart failure after myocardial infarction. Circulation.

[28] Fraccarollo D, Galuppo P, Bauersachs J (2012). Novel therapeutic approaches to post-infarction remodeling. Cardivasc Res.

[29] Furth R, Cohn ZA (1968). The origin and kinetics of mononuclear phagosytes. J Exp Med.

[30] Fujiu K, Wang J, Nagai R (2014). Cardioprotective function of cardiac macrophages. Cardiovasc Res.

[31] Perdiguero EG, Klapproth K, Schulz C, Busch K, Azzoni E, Crozet L (2015). Tissue-resident macrophages originate from yolk-sac-derived erythro-myeloid progenitors. Nature.

[32] Hoeffel G, Wang Y, Greter M, See P, Teo P, Malleret B (2012). Adult Langerhans cells derive predominantly from embryonic fetal liver monocytes with a minor contribution of yolk sac-derived macrophages. J Exp Med.

[33] Gosselin D, Link VM, Romanoski CE, Fonseca GJ, Eichenfield DZ, Spann NJ (2014). Environment drives selection and function of enhancers controlling tissue-specific macrophage identities. Cell.

[34] Murray PJ, Wynn TA (2011). Protective and pathogenic functions of macrophage subsets. Nat Rev Immunol.

[35] Schulz C, Perdiguero EG, Chorro L, Szabo-Rogers H, Cagnard N (2012). A lineage of myeloid cells independent of Myb and hematopoietic stem cells. Science.

[36] Bain CC, Bravo-Blas A, Scott CL, Gomez Perdiguero E, Geissmann F, Henri S (2014). Constant replenishment from circulating monocytes maintains the macrophage pool in the intestine of adult mice. Nat Immunol.

[37] Molawi K, Wolf Y, Kandalla PK, Favret J, Hagemeyer N, Frenzel K (2014). Progressive replacement of embryo-derived cardiac macrophages with age. J Exp Med.

[38] Schulz C, Massberg S (2014). Atherosclerosis – multiple pathways to lesional macrophages. Sc I Transl Med.

[39] Wynn TA, Chawla A, Pollard JW (2013). Macrophage biology in development, homeostasis and disease. Nature.

[40] Gratchev A, Kzhyshkowska J, Muller-Molinet I, Kannookadan S, Utikal J, Coerdt S (2006). Mphi1 and Mphi2 can be re-polarized by Th2 or Th1 cytokines, respectively, and respond to exogenous danger signals. Immunobiology.

[41] Mackaness GB (1969). The influence of immunologically committed lymphoid cells on macrophage activity in vivo. J Exp Med.

[42] Mantovani A, Sozzani S, Locati M, Allavena P, Sica A (2002). Macrophage polarization: tumor-associated macrophages as a paradigm for polarized M2 mononuclear phagocytes. Trends Immunol.

[43] Gordon S, Martinez FO (2010). Alternative activation of macrophages: mechanism and functions. Immunity.

[44] Biswas SK, Mantovani A (2010). Macrophage plasticity and interaction with lymphocyte subsets: cancer as a paradigm. Nat Immunol.

[45] Mantovani A, Sica A, Sozzani S, Allavena P, Vecchi A, Locati M (2004). The chemokine system in diverse forms of macrophage activation and polarization. Trends Immunol.

[46] Murray PJ, Allen JE, Biswas SK, Fisher EA, Gilroy DW, Goerdt S (2014). Macrophage activation and polarization: nomenclature and experimental guidelines. Immunity.

[47] Sica A, Mantovani A (2012). Macrophage plasticity and polarization: in vivo veritas. J Clin Invest.

[48] Mulder R, Banete A, Basta S (2014). Spleen-derived macrophages are readily polarized into classically activated (M1) or alternatively activated (M2) states. Immunibiol.

[49] Gordon S (2003). Alternative activation of macrophages. Nat Rev Immunol.

[50] Pesce J, Kaviratne M, Ramalingam TR (2006). The IL-21 receptor augments Th2 effector function and alternative macrophage activation. J Clin Invest.

[51] Hazlett LD, McClellan SA, Barrett RP, Thompson RW, Urban JF, Cheever AW (2010). IL-33 shifts macrophage polarization, promoting resistance against Pseudomonas aeruginosa keratitis. Invest Ophthalmol Vis Sci.

[52] Martinez FO, Gordon S, Locati M, Mantovani A (2006). Transcriptional profiling of the human monocyte-to-macrophage differentiation and polarization: new molecules and patterns of gene expression. J Immunol.

[53] Dinarello CA (2005). Blocking IL-1 in systemic inflammation. J Exp Med.

[54] Mantovani A (2008). From phagocyte diversity and activation to probiotics: back to Metchnikoff. Eur J Immunol.

[55] Medzhitov R, Horng T (2009). Transcriptional control of the inflammatory response. Nat Rev Immunol.

[56] Kzhyshkowska J, Gratchev A, Goerdt S (2006). Stabilin-1, a homeoststic scavenger receptor with multiple functions. J Cell Mol Med.

[57] MacLeod AS, Mansbridge JN. The Innate Immune System in Acute and Chronic Wounds. Adv Wound Care (New Rochelle). 2016;5:65-78. 10.1089/wound.2014.0608 PMC4742992 26862464

[58] Mantovani A, Biswas SK, Galdiero MR, Sica A, Locati M (2014). Macrophage plasticity and polarization in tissue repair and remodeling. J Pathol.

[59] Nahrendorf M, Swirski F (2013). Monocyte and macrophage heterogeneity in the heart. Circ Res.

[60] Lindsey M, Saucerman J, Deleon-Penell K (2016). Knowledge gaps to understanding cardiac macrophage polarization following myocardial infarction. Biochim Biophys Acta.

[61] Weiberger T, Schulz C (2015). Myocardial infarction: a critical role of macrophages in cardiac remodeling. Front Physiol.

[62] Frith JC, Monkkonen J, Blackburn GM, Russell RG, Rogers MJ (1997). Clodronate and liposome encapsulated clodronate are metabolized to a toxic ATP analog, adenosine 5′-(beta, gamma-dichloromethylene) triphosphate, by mammalian cells in vitro. J Bone Miner Res.

[63] an Amerongen MJ, Harmsen MC, van Rooijen N, Petersen AH, van Luyn MJ (2007). Macrophage depletion impairs wound healing and increases left ventricular remodeling after myocardial injury in mice. Am J Pathol.

[64] Ren W, Zhang R, Markel DC, Wu B, Peng X, Hawkins M (2007). Blockade of vascular endothelial growth factor activity suppresses wear debris-induced inflammatory osteolysis. J Rheumatol.

[65] Yan D, Wang X, Li D, Liu W, Li M, Qu Z (2013). Macrophages overexpressing VEGF target to infarcted myocardium and improve neovascularization and cardiac function. Int J Cardiol.

[66] Hilgendorf I, Gerhardt LM, Tan TC, Winter C, Holderried TA, Chousterman BG (2014). Ly-6Chigh monocytes depend on Nr4a1 to balance both inflammatory and reparative phases in the infarcted myocardium. Circ Res.

[67] Nahrendorf M, Swirski FK, Aikawa E, Stangenberg L, Wurdinger T, Figueiredo JL (2007). The healing myocardium sequentially mobilizes two monocyte subsets with divergent and complementary functions. J Exp Med.

[68] Woollard KJ, Geissmann F (2010). Monocytes in atherosclerosis: subsets and functions. Nat Rev Cardiol.

[69] Humeres C, Vivar R, Boza P, Muñoz C, Bolivar S, Anfossi R, et al. Cardiac fibroblast cytokine profiles induced by proinflammatory or profibrotic stimuli promote monocyte recruitment and modulate macrophage M1/M2 balance in vitro. J Mol Cell Cardiol. 2016. doi:10.1016/j.yjmcc.2016.10.014. 10.1016/j.yjmcc.2016.10.014 27983968

[70] Carlson S, Helterline D, Asbe L, Dupras S, Minami E, Farris S, et al. Cardiac macrophages adopt profibrotic/M2 phenotype in infarcted hearts: Role of urokinase plasminogen activator. J Mol Cell Cardiol. 2016. doi: 10.1016/j.yjmcc.2016.05.016. 10.1016/j.yjmcc.2016.05.016 27262672

[71] Miao L, Shen X, Whiteman M, Xin H, Shen Y, Xin X (2016). Hydrogen sulfide mitigates myocardial infarction via promotion of mitochondrial biogenesis-dependent M2 polarization of macrophages. Antioxid Redox Signal.

[72] Epelman S, Lavine KJ, Beaudin AE, Sojka DK, Carrero JA, Calderon B (2014). Embryonic and adult-derived resident cardiac macrophages are maintained through distinct mechanisms at steady state and during inflammation. Immunity.

[73] Shiraishi M, Shintani Y, Shintani Y, Ishida H, Saba R, Yamaguchi A (2016). Alternatively activated macrophages determine repair of the infarcted adult murine heart. J Clin Invest.

[74] Hanna RN, Carlin LM, Hubbeling HG, Nackiewicz D, Green AM, Punt JA (2011). The transcription factor NR4A1 (Nur77) controls bone marrow differentiation and survival of Ly6C-monocytes. Nat Immunol.

[75] Nahrendorf M, Pittet MJ, Swirski FK (2010). Monocytes: protagonists of infarct inflammation and repair after myocardial infarction. Circulation.

[76] Hasan AS, Luo L, Yan C, Zhang TX, Urata Y, Goto S (2016). Cardiosphere-derived cells facilitate heart repair by modulating M1/M2 macrophage polarization and neutrophil recruitment. PLoS One.

[77] Harel-Adar T, Mordechai TB, Amsalem Y, Feinberg MS, Leor J, Cohen S (2011). Modulation of cardiac macrophages by phosphatidylserine-presenting liposomes improves infarct repair. Proc Natl Acad Sci.

[78] Troidl C, Mollmann H, Nef H, Masseli F, Voss S, Szardien S (2009). Classically and alternatively activated macro- phages contribute to tissue remodelling after myocardial infarction. J Cell Mol Med.

[79] Ryabov VV, Kirgizova MA, Suslova TE, Poponina YS, Markov VA, Karpov RS (2014). Long-term clinical results of autologous bone marrow mononuclear cell transplantation in patients with acute myocardial infarction. Siberian Medical Journal, Tomsk.

[80] Markov VA, Ryabov VV, Maksimov IV, Vyshlov EV, Demyanov SV, Syrkina AG (2011). Yesterday, today, tomorrow in diagnostics and treatment of acute myocardial infarction. Siberian Medical Journal, Tomsk.

[81] Sinning C, Kempf T, Schwarzl M, Lanfermann S, Ojeda F, Schnabel RB (2017). Biomarkers for characterization of heart failure - distinction of heart failure with preserved and reduced ejection fraction. Int J Cardiol.

[82] Damman P, Kempf T, Windhausen F, an Straalen JP, Guba-Quint A, Fischer J (2014). Growth-differentiation factor 15 for long-term prognostication in patients with non-ST-elevation acute coronary syndrome: an Invasive versus Conservative Treatment in Unstable coronary Syndromes (ICTUS) substudy. Int J Cardiol.

[83] Mezzaroma E, Toldo S, Farkas D, Seropian IM, Van Tassell BW, Salloum FN (2011). The inflammasome promotes adverse cardiac remodeling following acute myocardial infarction in the mouse. Proc Natl Acad Sci U S A.

[84] Frangogiannis NG, Dewald O, Xia Y, Ren G, Haudek S, Leucker T (2007). Critical role of monocyte chemoattractant protein-1/CC chemokine ligand 2 in the pathogenesis of ischemic cardiomyopathy. Circulation.

[85] Zhao G, Wang S, Wang Z, Sun A, Yang X, Qiu Z (2013). CXCR6 deficiency ameliorated myocardial ischemia/reperfusion injury by inhibiting infiltration of monocytes and IFN-gamma-dependent autophagy. Int J Cardiol.

[86] Gao XM, Liu Y, White D, Su Y, Drew BG, Bruce CR (2011). Deletion of macrophage migration inhibitory factor protects the heart from severe ischemia-reperfusion injury: a predominant role of anti-inflammation. J Mol Cell Cardiol.

[87] Liu H, Wu X, Gang N, Wang S, Deng W, Zan L, et al. Macrophage functional phenotype can be consecutively and reversibly shifted to adapt to microenvironmental changes. Int J Clin Exp Med. 2015;8:3044-53. PMC4402928 25932281

[88] Weirather J, Hofmann UDW, Beyersdorf N, Ramos GC, Vogel B, Frey A (2014). Foxp3 + CD4-T cells improve healing after myocardial infarction by modulating monocyte/macrophage differentiation. Circ Res.

[89] Zouggari Y, Ait-Oufella H, Bonnin P (2013). B lymphocytes trigger monocyte mobilization and impair heart function after acute myocardial infarction. Nat Med.

[90] Hamada M, Nakamura M, Tran MT, Moriguchi T, Hong C, Ohsumi T (2014). MafB promotes atherosclerosis by inhibiting foam-cell apoptosis. Nat Commun.

[91] Jenkins SJ, Ruckerl D, Thomas GD, Hewitson JP, Duncan S, Brombacher F (2013). IL-4 directly signals tissue-resident macrophages to proliferate beyond homeostatic levels controlled by CSF-1. J Exp Med.

[92] Das A, Ganesh K, Khanna S, Sen CK, Roy S (2014). Engulfment of apoptotic cells by macrophages: a role of MicroRNA-21 in the resolution of wound inflammation. J Immunol.

[93] Das A, Sinha M, Datta S, Abas M, Chaffee S, Sen CK (2015). Monocyte and macrophage plasticity in tissue repair and regeneration. Am J Pathol.

[94] Lawrence T, Natoli G (2011). Transcriptional regulation of macrophage polarization: enabling diversity with identity. Nat Rev Immunol.

[95] Graff JW, Dickson AM, Clay G, McCaffrey AP, Wilson ME (2012). Identifying functional microRNAs in macrophages with polarized phenotypes. J Biol Chem.

[96] Roy S (2016). miRNA in macrophage development and function. Antioxid Redox Signal.

[97] Bauersachs J (2010). Regulation of myocardial fibrosis by MicroRNAs. J Cardiovasc Pharmacol.

[98] Small EM, Olson EN (2011). Pervasive roles of microRNAs in cardiovascular biology. Nature.

[99] Zhu H, Fan G (2011). Role of microRNA in reperfused myocardium towards post-infarct remodeling. Cardiovasc Res.

[100] Thum T, Galuppo P, Wolf C, Fiedler J, Kneitz S, van Laake LW (2007). MicroRNAs in the human heart: a clue to fetal gene reprogramming in heart failure. Circulation.

[101] Bauersachs J, Thum T (2011). Biogenesis and regulation of cardiovascular microRNAs. Circ Res.

[102] Roy S, Khanna S, Hussain SR, Biswas S, Azad A, Rink C (2009). MicroRNA expression in response to murine myocardial infarction: miR-21 regulates fibroblast metalloprotease-2 via phosphatase and tensin homologue. Cardiovasc Res.

[103] Dong S, Cheng Y, Yang J, Li J, Liu X, Wang X (2009). MicroRNA expression signature and the role of microRNA-21 in the early phase of acute myocardial infarction. J Biol Chem.

[104] Van Rooij E, Sutherland LB, Thatcher JE, DiMaio JM, Naseem RH, Marshall WS (2008). Dysregulation of microRNAs after myocardial infarction reveals a role of miR-29 in cardiac fibrosis. Proc Natl Acad Sci U S A.

[105] Bonauer A, Carmona G, Iwasaki M, Mione M, Koyanagi M, Fischer A (2009). MicroRNA-92a controls angiogenesis and functional recovery of ischemic tissues in mice. Science.

[106] Fiedler J, Jazbutyte V, Kirchmaier BC, Gupta SK, Lorenzen J, Hartmann D (2011). MicroRNA-24 regulates vascularity after myocardial infarction. Circulation.

[107] Schroen B, Heymans S (2012). Small but smart – microRNAs in the center of inflammatory processes during cardiovascular diseases, the metabolic syndrome, and aging. Cardiovasc Res.

